# Concentrations of total, bioavailable, and free 25OHD in individuals with and without primary hyperparathyroidism and their correlations to DXA and trabecular bone score

**DOI:** 10.20945/2359-3997000000529

**Published:** 2023-01-17

**Authors:** Lívia Marcela Santos, Monique Ohe, Sthefanie Pallone, Isabela Nacaguma, Ilda Kunii, Renata Silva, Cynthia Maria Brandão, José Gilberto Vieira, Marise Lazaretti-Castro

**Affiliations:** 1 Universidade Federal de São Paulo Escola Paulista de Medicina São Paulo SP Brasil Universidade Federal de São Paulo, Escola Paulista de Medicina, São Paulo, SP, Brasil

**Keywords:** 25-hydroxyvitamin D, trabecular bone score, bone mineral density, DBP polymorphism

## Abstract

**Objective:**

This study aimed to investigate the association between 25OHD (total, bioavailable and free) with bone mass and microarchitecture among primary hyperparathyroidism (PHPT) patients and controls.

**Subjects and methods:**

Sixty-four patients in the preoperative period of PHPT and 63 matched controls, who had not taken vitamin D in the last three months. To calculate the bioavailable and free 25OHD, the genetic variants of the vitamin D-binding protein (DBP) were determined. Bone mineral density (BMD) was determined by dual-energy X-ray absorptiometry (DXA). The distributions of total, bioavailable and free 25OHD and their correlation with TBS and DXA were evaluated.

**Results:**

PHPT showed BMD and TBS values lower than CTRL in all locations (p < 0.05). There were no statistical differences in the levels of free, bioavailable and total 25OHD between the PHPT and CTRL groups [mean, min-max: 3.4 (1.4-8.6) vs. 3.1 (1.0-9.8) pg/mL, 1.51 (0.43-3.58) vs. 1.41 (0.38-3.48) ng/mL, 22.6 (11.0-39.9) vs. 20.6 (8.9-35.3) ng/dL, respectively; (p > 0.05). The distribution of DBP haplotypes was similar between groups. DXA showed no correlation with any form of 25OHD in both groups. TBS presented a weak correlation with the total 25OHD in PHPT (r = 0.28; p = 0.02) and a moderate correlation with the total, free and bioavailable 25OHD in CTRL (r = 0.42; r = 0.42; r = 0.43; respectively, p < 0.01).

**Conclusion:**

The concentrations of total, free and bioavailable 25OHD were similar in both the PHPT and control groups. 25OHD concentrations correlated positively with TBS and not with DXA, especially in controls, suggesting that this method may be more sensitive to assessing the consequences of vitamin D deficiency on bone quality in individuals without PHPT.

## INTRODUCTION

The vitamin D molecule goes through a complex metabolic pathway, working as a potent regulator of bone and mineral homeostasis and presenting activities of differentiation, cellular growth, and immunomodulation on several other target tissues ( 1 ). Like all steroidal hormones, vitamin D circulates bound to a carrier protein – vitamin D binding protein (DBP), which in turn has a different affinity depending on its genetic variation ( *rs* 4588 and *rs* 7041) ( 2 ).

The DBP is a transport protein, which, along with albumin, bounds to more than 99% of 25OH vitamin D in the circulation. For most tissues, unbound 25OHD is the one that enters the cells and exercises its biological activity (free hormone hypothesis) but in some cells such as kidney, parathyroid, and placental cells, DBP participates in transporting 25OHD inside the cells through a megalin/cubilin complex ( 3 ).

Total 25OHD is the most commonly used studied form that provides the best measure of vitamin D status. Approximately 0.03% of 25OHD is in the free form (free vitamin D), 85% is bound to DBP, and 15% is to albumin ( 2 ). The bioavailable vitamin D designated by the sum of the free vitamin D with the one bound to albumin ( 4 ).

Even today the serum concentration of 25OHD is considered the gold standard to define the status of vitamin D in the individual. However, it is important to study other forms of vitamin D, mainly due to the free hormone hypothesis, which postulates that only the hormone not bound to its carrier protein is capable of exerting its biological activity. About DBP we know that some clinical situations can influence its serum concentration, some authors have dedicated themselves to studying the relationship between DBP and primary hyperparathyroidism ( 5 , 6 ).

Several authors have studied the correlation between BMD (bone mineral density) and 25OHD and, while some did not find a relationship between these factors ( 7 , 8 ), others showed a significant correlation between BMD and total 25OHD in specific groups such as menopausal women ( 9 ), adolescents ( 10 ), and adults ( 11 – 14 ).

There were major technological developments over the last years that also involved bone densitometry, resulting in image quality improvement and software enhancement, such as the Trabecular Bone Score (TBS), which assesses bone microarchitecture ( 15 ). The main role of this software is to help the prediction of fracture risk with the image of the lumbar spine obtained by bone densitometry. From this image, a texture assessment is performed, obtaining grey degrees and indirectly reflecting the integrity of trabecular microarchitecture ( 3 , 16 ). Moreover, TBS is a tool of easy application that may be obtained after performing bone densitometry and it is easily used in the clinical routine.

The relationship between TBS and vitamin D status is unclear in the literature. One of the first studies to investigate this relationship was performed in 2018, which verified those young and healthy Lebanese individuals with 25OHD higher than 30 ng/mL had a significantly higher TBS ( 17 ). Authors found a weak correlation between TBS and BMD, but TBS proved to be a good method for predicting fractures ( 18 ).

However, this tool is not used in daily clinical practice to assess PHPT ( 19 ). Primary hyperparathyroidism (PHPT) is a common endocrinopathy in clinical practice, characterized by hypercalcemia due to increased production of the parathyroid hormone (PTH) by one or more parathyroid glands. The improved availability of laboratory resources allowed changes in the clinical presentation of PHPT, with often asymptomatic patients ( 20 ). Therefore, the most usual signs and symptoms of PHPT are changes in bone tissue such as osteoporosis or fragility fractures. The association of 25OH vitamin D deficiency with the evolution of the bone status in PHPT has shown was described ( 21 ).

There is a consensus in the literature that the diagnosis of the majority of PHPT patients presents low vitamin D ( 22 ). Parathyroidectomy is the definite treatment in these cases but, before surgery, the medical conduct encouraged by consensus is the replacement of vitamin D to prevent potential bone hunger after surgery. This conduct, however, does not focus on preserving bone quality before surgery ( 23 ).

Some studies have shown a total, free, and bioavailable 25OHD deficiency in PHPT ( 24 , 25 ). This condition tends to lead to cases of fast and severe evolution and induces larger parathyroid adenomas ( 26 – 29 ). However, few studies correlate the different forms of 25OHD to bone quality in PHPT patients and CTRL.

This study aims to investigate the association between 25OHD (total, bioavailable and free) with bone mass and microarchitecture among HPTP patients and controls.

## PATIENTS AND METHODS

### Study design

This case-control study was performed at the Endocrinology Unit of the University Hospital of the Federal University of São Paulo, São Paulo, Brazil (Ethics Committee Report n. 0517/2018) and all participants signed an informed consent form before their inclusion in the study. This project was financially supported by Fapesp, n. 2017/19019-4 and CAAE (Certificate of Presentation for Ethical Appreciation): 89044117.9.0000.5505 and CEP/Unifesp: 0517/2018.

Individuals diagnosed with PHPT from June 2017 to January 2019, before parathyroidectomy, and individuals paired with controls without PHPT diagnosis were included.

The PHPT diagnosis was based on hypercalcemia associated with high or inappropriate PTH serum levels. The paired control group included volunteers of the community without PHPT diagnosis and who were not related to the affected individuals. The exclusion criteria for both groups were changes in renal function (creatinine depuration < 35 mL/min) calculated with the CKD-EPI (Chronic Kidney Disease Epidemiology Collaboration) equation, family history of PHPT, multiple endocrine neoplasia (MEN) syndrome, and familial hypocalciuric hypercalcemia (FHH). All participants should be not under cholecalciferol replacement for at least three months before starting the study. Postmenopause was defined as the absence of a menstrual period for more than one year.

### Laboratory tests

Venous blood samples were collected after night fasting, between 8 a.m. and 10 a.m., and maintained under refrigeration until their use. The samples were centrifuged at a low temperature (temperature of 4 °C and rotation of 2,000 rpm) and the serum and plasma were extracted and stored at -20 °C. Total calcium (tCa), ionized calcium (iCa), PTH, 25OHD, creatinine, phosphate (P), magnesium (Mg), alkaline phosphatase (AP), osteocalcin (OC), procollagen type 1 N-terminal propeptide (P1PN), and C-terminal telopeptides of type 1 collagen (CTX) were measured.

Bone turnover markers (P1NP, OC, and CTX) were assessed with commercial immunoassays (Chemiluminescence, Elecsys 2010 Analyzer; Roche Diagnostic, Indianapolis, IN, USA), according to the manufacturer's recommendations. For P1PN, the range considered normal was 13.8-60.9 ng/mL in postmenopausal women and 13.9-85.5 ng/mL in men, the intra-assay variation was 1.04%, and the inter-assay variation was 9.2%. For CTX, the range considered normal was < 0.650 ng/mL in women and < 0.850 ng/mL in men, the intra-assay variation was 3.1%, and the inter-assay variation was 3.4%. For OC, the normal range was 7.3-37.8 ng/mL in postmenopausal women and 10.8-31.1 ng/mL in men, the intra-assay variation was 0.7%, and the inter-assay variation was 1.0%.

The PTH, was measured with an immunometric assay (Roche, Elecsys Analyzer 2010, USA), according to the manufacturer's recommendations. The normality range was 10-65 pg/mL, intra-assay variation was 1.6%, and inter-assay variation was 2.1%. The iCa was measured with an ion-specific electrode (AVL 9180 Electrolyte Analyzer, AVL Scientific Corp., Roswell GA, USA). The DBP was measured with a polyclonal antibody ELISA kit (R&D Systems, Minneapolis, MN, USA), with an intra-assay variation of 6.0% and inter-assay variation of 7.2%, which can recognize all DBP subtypes.

The tCa, albumin, and creatinine with automatic assays of the same standard, as well as 25OHD were measured with a chemiluminescence assay (Roche, Elecsys 2010 Analyzer, USA). The normal reference value was greater than 30 ng/mL, intra-assay variation was 8.5%, and inter-assay variation was 9.2%.

### Bone densitometry

Bone mineral density (BMD) was measured with dual-energy X-ray absorptiometry (DXA; Hologic QDR 4500, Waltham, MA, USA) in the lumbar spine L1-L4 (LS), total femur (TF), femoral neck (FN), and 33% radius (R33). The body composition of the participants was also analyzed. The examination was performed in the first appointment. The values of BMD, Z-Score, and T-Score were considered for each site. The least significant change (LSC) in our service was the measurements of BMD in the lumbar spine, total hip, and femoral neck were 3.5%, 3.8%, and 3.8%, respectively.

### Trabecular Bone Score (TBS)

The TBS analyses were performed at the LS site analyzed by bone densitometry, using the TBS obtained from iNsight™ Software (v.3.0, Medimaps Group SA, Switzerland). The TBS calculates the mean value of individual measurements for the lumbar vertebrae (L1-L4). The present project considered a TBS score ≥ 1.310 consistent with low fracture risk, corresponding to the normal microarchitecture; TBS values between 1.230 and 1.310 consistent with partially degraded microarchitecture; and TBS ≤ 1.230 defined as degraded microarchitecture with high fracture risk, as described by McCloskey and colleagues ( 30 ).

### Total DNA extraction and DBP genotyping

The DBP genotype analysis was performed with peripheral leukocytes of PHPT patients and controls, and commercial kits were used according to the manufacturer's recommendations (Gentra Puregene Blood Kit, PUREGENE, QIAGEN) or an in-house protocol ( 31 ). The total DNA was quantified by measuring absorbance at 260 nm in a spectrophotometer (NanoVue Plus GE Healthcare, Buckinghamshire, UK).

Genotyping for *rs* 4588 and *rs* 7041 was performed with a real-time polymerase chain reaction (PCR) (Applied Biosystems, California, USA), using the allelic discrimination assay with TaqMan MGB primers and probes (Applied Biosystems, California, USA). The DBP isoforms were built from the combination of *rs* 4588 and *rs* 7041 polymorphisms, initially classified as Gc1f/1f, Gc1f/1s Gc1s/1s, Gc1f/2, Gc 1s/2, and Gc2/2.

### Bioavailable and free vitamin D calculation

Free vitamin D calculation is based on the concentration values of DBP, albumin, and 25OHD. The DBP genotype was used to correct the affinity constant (Ka) according to Gc ( 32 ). The Ka of DBP was used 1s/1s: 0.6x10^9^ M^−1^, 1f/1f: 1.12x10^9^ M^−1^, 1s/1f: 0.86x10^9^ M^−1^, 1s/2: 0.48x10^9^ M^−1^, 1f/2: 0.74x10^9^ M^−1^, and 2/2: 0.36x10^9^ M^−1^.

### Statistical analysis

The Shapiro-Wilk normality test was used. The quantitative data are presented as mean ± standard deviation (SD), median, minimum and maximum, or percentage. No parametric tests were used to analyze non-Gaussian variables. Kruskal-Wallis was used to compare the means of the groups. Pearson's chi-square test (χ2) was used to test categorical variables. The test used for correlation was the Spearman. All analyses were performed in the GraphPad Prism Statistics, version 8.0.1 (Chicago, IL, USA). The values of p < 0.05 were considered significant.

## RESULTS

### Demographics and clinical characteristics

Sixty-four patients with PHPT and 63 controls were analyzed, according to the criteria aforementioned.

For the age, nephrolithiasis, osteoporosis, bisphosphonate, vertebral fracture, total fracture, menopause and BMI parameters the Mann-Whitney test was used. For the parameters gender and Fitzpatrick the chi-square test was used.

The groups were similar for sex, age, BMI, menopause, and skin color distribution (Fitzpatrick). However, the PHPT group had a higher number of individuals with nephrolithiasis, previous or current use of bisphosphonate, and osteoporosis ( Table 1 ).

**Table 1:** Demographic and clinical aspects of patients with primary hyperparathyroidism (PHPT) and controls (CTRL) who performed bone densitometry

Clinical description	PHPT (n = 64)	CTRL (n = 63)	p-value
Male n (%)	9 (14.1)	14 (22.2)	0.26
Female n (%)	55 (85.9)	49 (77.8)	
Age (years) mean ± SD	66 ± 6.8	61 ± 7.6	0.11
Nephrolithiasis n (%)	24 (37.5)	9 (14.3)	<0.01
Osteoporosis n (%)	32 (50)	18 (28.6)	0.02
Bisphosphonate n (%)	22 (34.4)	5 (7.9)	<0.01
Vertebral fracture n (%)	9 (14.1)	4 (6.4)	0.02
Total fracture n (%)	15 (23.4)	11 (17.5)	0.51
Menopause n (%)	50 (90.9)	46 (93.9)	0.42
Fitzpatrick I, II, and III n (%)	38 (59.4)	38 (60.3)	0.99
Fitzpatrick IV, V, and VI n (%)	26 (40.6)	25 (39.7)	
BMI (kg/m^2^) mean ± SD	28.8 ± 5.12	28.5 ± 5.78	0.41

BMI: body mass index; SD: standard deviation; n: number of participants.

Regarding fractures, the distribution included the following: nine fractures in PHPT patients are vertebral, four forearm fractures, and two appendicular fractures (foot and hand). In the control group, there were four vertebral, one forearm, one femoral neck, and five appendicular fractures.

### Laboratory characteristics

Table 2 presents the biochemical data of PHPT patients and CTRL, with higher levels of PTH, tCa, iCa, P, AP, OC, and CTX in PHPT patients, as expected. The concentrations of P1NP; total, bioavailable, and free 25OHD; magnesium; and creatinine were not different between the groups.

**Table 2:** Laboratory parameters of patients with primary hyperparathyroidism (PHPT) and controls (CTRL) who performed bone densitometry

Median (min-max)	PHPT (n = 64)	CTRL (n = 63)	p
tCa (mg/dl)	11.25 (10.3-14.1)	9.7 (8.5-10.3)	<0.001
iCa (mmol/L)	1.48 (1.31-1.89)	1.29 (1.10 -1.35)	<0.001
PTH (pg/mL)	140.9 (53.3-558.8)	58.7 (15.6-101.1)	<0.01
P (mg/dL)	2.85 (2.0-4.9)	3.6 (2.3-4.8)	<0.001
ClCr (mL/kg/min)	71.7 (49-120)	77.5 (48-125)	0.50
AP (U/L)	86 (46-410)	70 (35-219)	0.005
OC (ng/mL)	30.4 (7.3-193.1)	17.4 (8.4-32.9)	<0.001
P1NP (ng/mL)	66.0 (13.6-70.5)	62.2 (20.4-115.1)	0.42
CTX (ng/mL)	0.45 (0.09-1.49)	0.31(0.11-0.75)	0.004
DBP (μg/mL)	268.6 (143.3-703.4)	274.7 (160.5-704.0)	0.60
25OHD (ng/mL)	22.6 (11.0-39.9)	20.6 (8.9-35.3)	0.13
Bio 25OHD (ng/mL)	1.51 (0.43-3.58)	1.41(0.38-3.48)	0.28
Free 25OHD (pg/mL)	3.4 (1.4-8.6)	3.1 (1.0-9.8)	0.44

PHPT: primary hyperparathyroidism; CTRL: control; RV: reference value; tCa: total calcium; iCa: ionized calcium; PTH: parathyroid hormone; P: phosphorus; Cr: creatinine; AP: alkaline phosphatase; 25OHD: vitamin D; CTX: C-terminal telopeptides of type 1 collagen; OC: osteocalcin; P1NP: procollagen type 1 N-terminal propeptide.

The Mann-Whitney statistical test was used. Values were considered significant if p < 0.05.

### Densitometric parameters

Table 3 shows that PHPT patients presented lower statistically significant BMD values in all sites assessed in comparison to CTRL. The same was observed for TBS measures.

**Table 3:** Results obtained by bone densitometry, including bone mineral density (BMD), T-score, and TBS of both groups. Presented as mean ± standard deviation

Parameters	PHPT (n = 64) mean ± SD	CTRL (n = 63) mean ± SD	p
TBS	1.233 ± 0.130	1.280 ± 0.130	0.04
LS-BMD (g/cm^2^)	0.892 ± 0.14	0.985 ± 0.17	0.001
LS T-score	-1.5 ± 1.3	-0.7 ± 1.5	0.004
FN-BMD (g/cm^2^)	0.695 ± 0.09	0.812 ± 0.14	<0.001
FN T-score	-1.4 ± 0.8	-0.5 ± 1.2	<0.001
TF-BMD (g/cm^2^)	0.806 ± 0.11	0.916 ± 0.16	<0.001
TF T-score	-1.2 ± 0.9	-0.4 ± 1.0	0.001
R33- BMD (g/cm^2^)	0.570 ± 0.1	0.657 ± 0.09	<0.001
R33 T-score	-2.4 ± 1.6	-1.1 ± 1.2	<0.001

TBS: trabecular bone score; LS-BMD: bone mineral density in the lumbar spine; FN-BMD: bone mineral density in the femoral neck; TF-BMD: bone mineral density in the total femur; R33-BMD: bone mineral density in the 33% radius.

Osteoporosis diagnosis by bone densitometry in at least one site analyzed (LS, TF, FN, and/or R33) was observed in 50% of PHPT patients and 28.6% of CTRL, osteopenia was found in 40.8% of PHPT patients and 38.1% of CTRL, and normal in 9.2% of PHPT patients and 33.9% of CTRL (p = 0.002).

**Figure 1:**
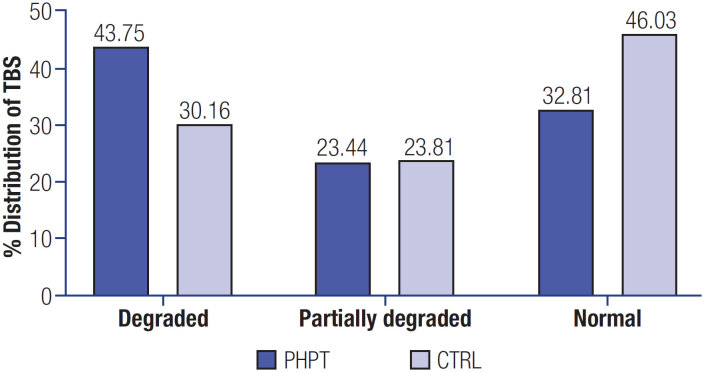
Distribution of TBS's defined bone structures between PHPT and control groups.

In figure 1 it observes the TBS distribution in the groups shows that degraded TBS (<1.230) is present in 43.75% (n = 28) of PHPT patients and 30.16% (n = 19) of CTRL, partially degraded TBS (TBS between 1.231 and 1.309) is found in 23.4% (n = 15) of PHPT patients and 23.8% (n = 15) of CTRL, and normal TBS (>1.310) is found in 32.8% (n = 21) of PHPT patients and 46% (n = 29) of CTRL, with no statistically significant differences between the groups (p = 0.08).

### Correlation between Trabecular Bone Score (TBS) and laboratory parameters

**Figure 2:**
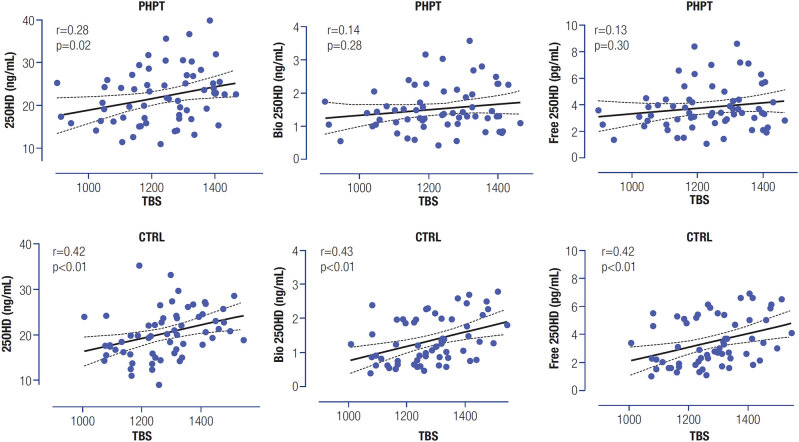
Graphs of the correlation between total, bioavailable, and free 25OHD and TBS in the groups of patients with primary hyperparathyroidism (PHPT) and controls (CTRL).

The correlation is higher in CTRL than in PHPT groups. In CTRL, all forms of vitamin D (total, bioavailable, and free 25OHD) correlate to TBS, with a moderate correlation (r = 0.42; r = 0.43; r = 0.42; respectively). The PHPT group shows a weak correlation (r = 0.28) only to 25OHD total ( Figure 2 ).

### Correlation between densitometry and laboratory parameters

Table 4 shows the lack of correlation of T-Score in all densitometry sites to the forms of vitamin D.

**Table 4:** Correlation between T-score in the lumbar spine (LS), femoral neck (FN), and total femur (TF) and laboratory parameters

Densitometric sites	PHPT		CTRL	
LS T-score	r	p	r	p
25OHD (ng/mL)	0.21	0.10	0.20	0.13
Bioavailable 25OHD (ng/mL)	0.15	0.25	0.23	0.09
Free 25OHD (pg/mL)	0.17	0.18	0.22	0.10
**TF T-score**				
25OHD (ng/mL)	-0.10	0.45	0.11	0.39
Bioavailable 25OHD (ng/mL)	0.06	0.66	0.11	0.42
Free 25OHD (pg/mL)	0.11	0.41	0.09	0.52
**FN T-score**				
25OHD (ng/mL)	0.11	0.40	0.06	0.64
Bioavailable 25OHD (ng/mL)	0.11	0.41	-0.01	0.95
Free 25OHD (pg/mL)	0.17	0.19	-0.03	0.81

PHPT: primary hyperparathyroidism; CTRL: control; RV: reference value; LS: lumbar spine; FN: femoral neck; TF: total femur.

### Distribution of DBP genotyping

The analysis of single nucleotide polymorphisms (SNPs) of DBP ( *rs* 4588 and *rs* 7041) did not show statistical differences between the PHPT and CTRL groups, with p = 0.93 and p = 0.76, respectively. Regarding the combination of these SNPs, meaning Cg 1f-1f, 1f-1s, 1fs-1s, 1f-2, 1s2, and 2-2, there were also no differences between the groups. In the comparison of the distribution of Gc 1-1, Gc 1-2, and Gc 2-2, there were also no differences between the groups, with p = 0.96.

## DISCUSSION

In this study, total, free and bioavailable 25OHD measurements didn’t present statistical differences between PHPT patients and controls. Regarding DBP genotype, some authors ( 33 , 34 ), as the authors of the present study, show the frequency of two common single nucleotide polymorphisms (SNPs) ( *rs* 7041 and 4588) in DBP were not distributed differently among PHPT patients compared to the control group. The SNPs distribution in the Brazilian population was similar to the distribution-related previously in the analysis of the southern Brazilian population ( 35 ).

Few studies aim for biochemical correlations with bone parameters among PHP patients. While some studies examine the correlation between 25OHD and DXA, with frequent findings of weak correlation among PHP patients ( 21 , 34 ), other reports observe that TBS, used as a complement to DXA measurements, may better access fragility fracture risk in PHPT patients ( 21 , 36 ). Nevertheless, the PHPT population has been rarely studied regarding TBS data and its correlations with the forms of 25OHD.

The concentration of 25OHD correlates to bone quality or even the ability to predict fractures ( 37 , 38 ). Low levels of 25OHD were associated with a higher risk of hip fracture and this association did not depend on falls, physical condition, fragility, renal function, or the use of steroidal hormones ( 37 ). Low levels of 25OHD are usually observed in primary hyperparathyroidism patients and some studies, as reported by Battista and cols. ( 33 ), describe that the calculated levels of free and bioavailable 25OHD were lower in PHPT patients than in controls (healthy family members sharing DBP with similar polymorphisms). Nevertheless, these data may not be extended to all PHPT patients, once individuals from other populations may present different clinical profiles ( 39 ) and different DBP genotypical distribution ( 40 ).

This study, as far as we are concerned, is the first to evaluate total, free and bioavailable 25OHD among PHPT patients in Brazil and correlations between total 25OHD and TBS in PHPT. Demonstrating a correlation of bone microarchitecture not only with total of 25OHD but also with the free forms (free and bioavailable 25OHD). We observed a positive correlation of TBS measurements to all forms of 25OHD among the control group, with a mean correlation of 0.40. On the other hand, in the PHPT group, this correlation was weaker and only with total 25OHD, demonstrating that the underlying disease overlaps the effects of vitamin D status on bone structure.

Some authors ( 33 , 41 ) also found lower serum concentration of DBP in HPTP compared to controls, several theories are postulated to explain this phenomenon, but the theory that PTH can act by regulating the concentration of DBP is the most accepted ( 41 , 42 ).

BMD values in all densitometric sites assessed were statistically lower in PHPT patients than in the control group ( Table 3 ) as expected, and BMD measurements by DXA showed no correlations with any form of 25OHD at any site in both groups. In addition, 25OHD appears to have a positive effect on bone microarchitecture evaluated by TBS, once this parameter was positively correlated with all three forms of 25OHD (total, bioavailable and free) in the control group.

Some limitations in our study should be considered. A single measurement of 25OHD levels was taken in each patient from both groups, which could not reflect the vitamin D status in the long term for this population. Moreover, the study included a limited number of participants. Additionally, the analysis of bone turnover markers may be interfered with by the use of bisphosphonate in both groups.

In conclusion, the concentrations of total, free and bioavailable 25OHD were similar in both the PHPT and control groups, and no differences in the distribution of DBP genotypes between PHPT and controls were found.

Within the population of our study, 25OHD (total, bioavalilable and free) concentrations correlated positively with TBS and not with DXA, especially in controls, suggesting the hypothesis that this method, TBS, may be more sensitive to assessing the consequences of vitamin D deficiency on bone quality in vitamin D insufficiency individuals without PHPT. Other studies must be carried out to confirm this hypothesis.
